# Role of endoplasmic reticulum stress in cell apoptosis induced by duck hepatitis A virus type 1 infection

**DOI:** 10.3389/fimmu.2025.1567540

**Published:** 2025-03-12

**Authors:** Jingjing Lan, Ruihua Zhang, Guige Xu, Hui Yan, Jingyu Wang, Xingxing Shi, Yanli Zhu, Zhijing Xie, Shijin Jiang

**Affiliations:** ^1^ College of Veterinary Medicine, Shandong Agricultural University, Taian, Shandong, China; ^2^ Shandong Provincial Key Laboratory of Zoonoses, Shandong Agricultural University, Tai'an, Shandong, China

**Keywords:** DHAV-1, ERS, UPR, CHOP, apoptosis

## Abstract

The endoplasmic reticulum (ER), an elaborate cellular organelle that interweaves the cytosol, nucleus, mitochondria and plasma membrane, is essential for cell function and survival. Disruption of ER function can trigger unfolded protein response (UPR), which is activated by ER stress (ERS). In this study, we investigated the role of ERS in cell apoptosis induced by duck hepatitis A virus type 1 (DHAV-1) infection. Our findings revealed that DHAV-1 infection led to the activation of ERS. Specially, the expression of glucose-regulated protein 78 (GRP78) was upregulated, activating two pathways of UPR: the protein kinase R-like ER kinase (PERK) pathway and the inositol-requiring enzyme 1(IRE1) pathway. Consequently, phosphorylation of eukaryotic initiation factor 2 alpha (p-eIF2α) was increased, and transcription factor 4 (ATF4) was up-regulated, resulting in the induction of the apoptotic C/EBP homologous protein (CHOP). DHAV-1-infected cells exhibited various apoptotic phenotypes, including growth arrest, induction of the DNA damage-inducible protein 34 (GADD34), activation of caspase-3, and suppression of antiapoptotic protein B cell lymphoma-2 (Bcl-2). Importantly, inhibition of PERK or protein kinase R (PKR) activity suppressed CHOP activation and DHAV-1 replication, indicating that the PERK/PKR-eIF2α pathway played a crucial role in ERS-induced apoptosis. Collectively, our study provides novel insights into the mechanism of DHAV-1-induced apoptosis and reveals a potential defense mechanism against DHAV-1 replication.

## Introduction

Duck viral hepatitis (DVH) is a highly contagious and acute disease that poses a significant threat to ducklings, with a high mortality rate, significantly impacting the poultry industry (1). The main pathogen responsible for DVH is duck hepatitis A virus (DHAV), which belongs to the *Avihepatovirus* genus in *Picornaviridae* family. DHAV consists of three genetically distinct genotypes and serotypes: DHAV-1, DHAV-2 and DHAV-3 (2–4). DHAV-1, being the classical and most prevalent serotype, is responsible for highly contagious and acute disease outbreaks in ducklings worldwide (5–8). Besides rapid horizontal transmission, which causes severe mortality in ducklings younger than 3 weeks of age, DHAV-1 can also infect laying ducks, causing egg drop syndrome (9). Furthermore, it can be vertically transmitted from breeding ducks to ducklings (10).

The endoplasmic reticulum (ER) is a highly intricate and dynamic organelle with diverse functions, including cellular metabolism and protein synthesis, folding, modification and trafficking. Endoplasmic reticulum stress (ERS) occurs when there is an accumulation of unfolded or misfolded proteins, activating the unfolded protein response (UPR) (11). The replication of viruses is closely associated with ER membranes, which serve as center sites for viral encapsulation and envelopment (12–15). During viral replication, many viral proteins, particularly glycoproteins, are synthesized for virus replication and maturation. The increased demand for proteins triggers ERS, leading to both cell survival and cell death.

Cell apoptosis is a complex regulatory network influenced by various factors, and viruses can induce cell apoptosis through multiple mechanisms, such as ERS, DNA damage, and disruption of Ca^2+^ homeostasis (16–20). Apoptosis has been recognized as a programmed cell death mechanism that facilitates virus spread during virus infection (21). Several viruses in the *Picornaviruses* family, such as foot and mouth disease virus, enterovirus, and poliovirus, have been reported to be associated with apoptosis (22–24). Studies have indicated that apoptosis may also play a significant role in the infection process of DHAV-1*in vitro* (25).

ERS-induced apoptosis has been implicated in the infection of various viruses, such as bovine viral diarrhea virus (BVDV) in animals and hepatitis C virus (HCV) in human (26, 27). Short-term activation of UPR helps cells adapt to ERS and maintain cellular homeostasis. However, persistent ERS and viral infections can induce cell apoptosis by regulating different apoptosis associated factors. One crucial factor is the transcription factor C/EBP homologous protein (CHOP), also known as growth arrest and DNA damage-inducible gene 153 (GADD153). Activation of CHOP can upregulate the pro-apoptosis protein GADD34 and downregulation the antiapoptotic protein Bcl-2 (28–30).

In the UPR pathway, glucose-regulated protein 78 (GRP78) acts as an ER chaperone and interacts with protein kinase R (PKR)-like ER kinase (PERK) to activate transcription factor 6 (ATF6) and inositol-requiring enzyme 1 (IRE1), keeping them in an inactive state under normal conditions (31–33). During ERS, misfolded proteins bind to GRP78, leading to the dissociation of cellular factors from GRP78. It has been reported that all three transmembrane proteins (PERK, ATF6 and IRE1) can induce CHOP transcription, and the eukaryotic initiation factor 2 alpha-ATF4 (PERK-eIF2α) pathway is crucial for to CHOP expression (34). During the early stage of UPR, PERK is released from GRP78 and undergoes self-phosphorylation, activating its kinase activity. The PERK-mediated phosphorylation of eIF2α (p-eIF2α) attenuates global protein translation and reduces protein folding load (35). P-eIF2α preferentially translates ATF4 mRNA and upregulates ATF3 transcription, leading to elevated CHOP expression. It has been reported that CHOP is responsible for inducting death receptor 5, contributing to ERS-induced apoptosis (36).

Viral infections often trigger ERS within the host cells, employing various strategies to manipulate this stress response for their own replication. However, the precise mechanisms by which DHAV-1 modulates the UPR and ERS-induced apoptosis remain unclear. This study aimed to investigate the impact of DHAV-1 infection on ERS and apoptosis, which will provide a novel insight into the apoptosis mechanisms induced by DHAV-1 infection contributing to our understanding of DHAV-1 pathogenesis.

## Materials and methods

### Virus and antibodies

The DHAV-1 LY0801 strain (accession no. FJ436047), a virulent strain (5), was propagated in 10-day-old specific-pathogen free (SPF) embryonated eggs for several days. The purified viral allantoic fluid was store at -80°C as the virus stock. Anti-DHAV-1 monoclonal antibody (mAb) 4F8, which binds to the liner epitope “^75^GEIILT^80^” in the VP1 protein of DHAV-1, was stored in our Laboratory (37). Antibodies against GRP78, PERK, p-PERK, eIF2α, p-eIF2α, *ATF4, CHOP* and *β-actin* were purchased from Cell Signaling Technologies. Antibodies against Bcl-2, ATF3, and GADD34 were purchased from Abcam. Anti-His tag antibody was purchased from Sigma. The horseradish peroxidase (HRP)-conjugated goat anti-mouse or goat anti-rabbit IgG antibody was obtained from Beyotime.

### Cells treatment

Duck embryo fibroblast (DEF) cells were prepared from 10-day-old SPF embryos purchased from the State Resource Center of Laboratory Animal for Poultry (Harbin, China). Baby hamster Syrian kidney-21 (BHK-21) cells were purchased from the American Type Culture Collection (ATCC) (Manassas, VA, USA). DEF and BHK-21 cells were maintained in standard Dulbecco’s modified Eagle’s medium (DMEM) supplemented with 10% fetal bovine serum (FBS) and grown at 37°C in 5% CO_2_. When the cells reached approximately 80%~90% confluence, they were infected with DHAV-1 at a multiplicity of infection (MOI) of 2.0. After adsorption for 2 h at 37°C, the medium was removed, cells were washed three times with phosphate-buffered saline (PBS), and fresh medium with 2% FBS was added. Mock-infected cells were used as a negative control. In the drug treatment groups, cells were treated with 2.5 μg/mL tunicamycin (Solarbio, Beijing, China), 3 mM 4-phenylbutyric acid (4-PBA) (Sigma, St. Louis, MO, USA), 4nM rapamycin (RAPA, Sigma, St. Louis, MO, USA), 1μM GSK2606424 (MedChem Express, NJ, USA), or 10 mM 2-AP (Sigma, St. Louis, MO, USA), respectively. All cells were cultured at 37°C under 5% CO_2_ until they were harvested at 12 to 72 hours post-infection (hpi).

### Real-time quantitative polymerase chain reaction analysis

Total RNA was extracted from mock-infected or DHAV-1 infected cells using TRIzol reagent (Promega, Madison, WI, USA) according to the manufacturer’s instructions. Two micrograms of the total RNA were then reverse transcribed using PrimeScript™ RT reagent Kit (TaKaRa, Dalian, China). RT-qPCR was carried out using the UltraSYBR mixture (TaKaRa, Dalian, China), with an initial denaturation step at 95°C for 5 min, followed by 40 cycles of denaturation at 95°C for 15 s and annealing and extension at 60°C for 34 s. The expression levels of *ATF4*, *CHOP* and *β-actin* were quantitative analyzed. The primer sequences used as follows: *ATF4* forward 5’-AAA GAA AAC TGG AGG GCC CC-3’ and reverse 5’-GTA GGA GTC TGG GCT CAT GC-3’, *CHOP* forward 5’-GGA GTG GCA GTG TTC CAG AG-3’ and reverse 5’-TGT TCA TCC TCA GTG CCC AC-3’, and *β-actin* forward 5’-GGT ATC GGC AGC AGT CCT A-3’ and reverse 5’-TTC ACA GAG GCG AGT AAC TT-3’. The relative expression levels of these genes were normalized to *β-actin* using the comparative cycle threshold (Ct) method (2^-△△Ct^).

### Western blotting analysis

Mock-infected and DHAV-1 infected cells were harvested at the indicated time points using the RIPA Lysis and Extraction Buffer (Invitrogen, Carlsbad, CA, USA). The lysates were centrifuged at 10,000 rpm for 10 min at 4°C to pellet cell debris, and the supernatants were collected. The protein concentrations were determined using the BCA kit (Beyotime, Shanghai, China). Equal amounts of total cell protein were separated on 7.5%~15% sodium dodecyl sulfate-polyacrylamide gel (SDS-PAGE) and transferred onto nitrocellulose (NC) membranes (Millipore Corp., Bedford, MA, USA) for western blotting analysis. Briefly, the membranes were blocked with 5% bovine serum albumin (BSA) blocking buffer for 1 h at room temperature, incubated with specific primary antibodies at 4°C overnight. After being washed three times with tris-buffered saline with 0.1% Tween 20 (TBST), the signals were developed by incubating with HRP-conjugated anti-rabbit or anti-mouse antibodies, visualized using the enhanced chemiluminescence substrate (ECL, Beyotime, Shanghai, China) according to the manufacturer’s protocol, and quantitated using Image J software.

### Observation with transmission electron microscope

DEF cells infected with DHAV-1 were grown in plates and collected at 48 hpi. Ultra-thin sections of the cells were observed using a H-7650 TEM (Hitachi, Tokyo, Japan). The morphology of ER membranes was observed as previously described (38). Mock cells were used as the negative control for comparison.

### Detection of X-box binding protein-1 splicing

Total RNA was isolated from DHAV-1-infected DEF cells, and cDNA was synthesized as mentioned above. The *XBP1* gene was amplified by RT-PCR using primers 5’-CGG GAC AGG AAG AAA GCG-3’ and 5’-ATT AAT GGC CTC CAG CTT AG-3’. The PCR products were subsequently digested with the restriction enzyme *Pst* I (Thermo Fisher Scientific, San Jose, CA, USA) and separated on a 1% agarose gel. As an internal control, *GAPDH* mRNA was amplified using the primers 5’-AGA TGC TGG TGC TGA ATA CG-3’ and 5’-ACT GTC TTC TGT GTG GCT GT-3’. Mock cells were used as the negative control.

### Annexin V-FITC and PI staining analysis

To analyze the level of apoptosis, 1×10^6^ cells were collected by centrifugation at 1000 rpm for 5 min and washed with PBS for three times. The cells were then resuspended in 1×binding buffer and incubated with FITC-conjugated Annexin V. Subsequently, cells were stained with propidium iodide (PI) according to the manufacturer of Annexin V-FITC/PI apoptosis kit (BD Bioscience, San Jose, CA, USA). The apoptosis cells were analyzed using flow cytometry. Mock cells were incubated as a negative control. Each experiment was performed in triplicate.

### Nuclear morphologic change

To detect DNA fragmentations during cell apoptosis, DHAV-1-infected cells were washed with PBS and fixed with cold methanol/acetone (1:1, *v*/*v*) for 5 min at room temperature. After removal of the fixation solution, the cells were washed three times with PBS and then stained with 2 μM Hoechst 33342 (Sigma-Aldrich, St. Louis, MO, USA) for 10 min at room temperature. The nuclear changes were observed under a fluorescence microscope (Olympus, Tokyo, Japan).

### DNA ladder analysis

DEF cells infected with DHAV-1 were harvested at 72 hpi by centrifugation. Total DNA was extracted with a DNA ladder extraction kit (Beyotime, Shanghai, China) and separated on a 1.5% agarose gel. The fragmented DNA bands were observed under the image analyzer (Syngene, Cambridge, UK).

## Results

### DHAV-1 infection activates ERS

**Figure 1:**
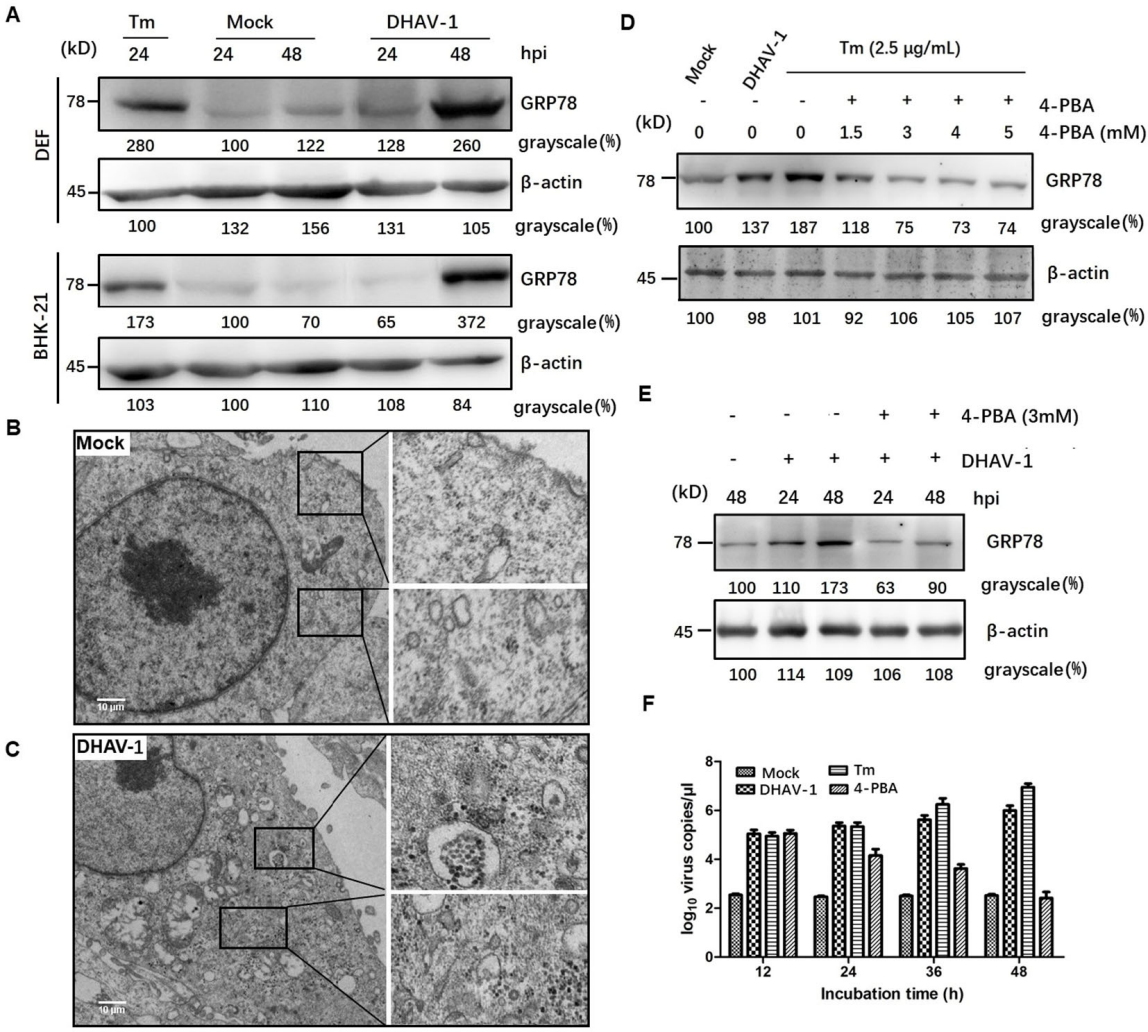
DHAV-1 infection activated the ERS. **(A)** DHAV-1 induces the GRP78 expression in DEF cells and BHK-21 cells. Tunicamycin (Tm) was respectively used as positive controls. **(B)** The ER ultrastructure in mock-infected DEF cells under transmission electron microscope (TEM) was used as the negative control. **(C)** The ER ultrastructure in DHAV-1-infected DEF cells revealed significant swelling under TEM. **(D)** The expression level of GRP78 showed a significant decrease upon 4-PBA treatment, with an optimal concentration of 3 Mm. **(E)** With 3 mM 4-PBA treatment, the induction of GRP78 was significantly lower at both 24 and 48 hpi. **(F)** Inhibition ERS with 4-PBA effectively reduced the replication of DHAV-1.

To investigate the initiation of ERS response during DHAV-1 infection, we examined the expression levels of GRP78, an ERS marker protein, through western blotting analysis. Both DEF cells and BHK-21 cells showed increased levels of GRP78 compared to the mock-infected cells (**Figure 1A**), similar to that in the cells treated with tunicamycin, which was used as a positive control. Interestingly, DEF cells exhibited higher GRP78 expression than BHK-21 cells at 24 hpi, potentially attributed to the greater proliferation efficiency of DHAV-1 in DEF cells. Contrasting with the normal appearance in mock-infected cells (**Figure 1B**), TEM revealed significant swelling of the ER membrane in DHAV-1-infected cells (**Figure 1C**). These findings strongly indicated that DHAV-1 infection activated ERS and UPR.

4-PBA has been demonstrated as an inhibitor of ERS (39). In this study, we utilized 4-PBA to mitigate ERS induced by DHAV-1 infection. Our result showed a significant decrease in the expression level of GRP78 upon 4-PBA treatment, with an optimal concentration of 3 mM (**Figure 1D**). Furthermore, at both 24 and 48 hpi, the induction of GRP78 was significantly lower with 3 mM 4-PBA treatment (**Figure 1E**).

During viral infection, numerous viral proteins are synthesized and accumulated in the ER lumen, exploiting the host’s translation machinery. For example, viruses such as HCV and African swine fever virus (ASFV) utilize ER as their replication sites to enhance virus replication (40, 41). To investigate the role of ERS in DHAV-1 replication, we quantified the number of viral mRNA copies in cells infected with DHAV-1 infection, as well as DHAV-1-infected cells with tunicamycin or 4-PBA treatment using RT-qPCR. The result showed that ERS contributed to DHAV-1 replication, and inhibition ERS with 4-PBA effectively reduced the replication of DHAV-1 (**Figure 1F**).

### DHAV-1 infection upregulates eIF2a phosphorylation

**Figure 2:**
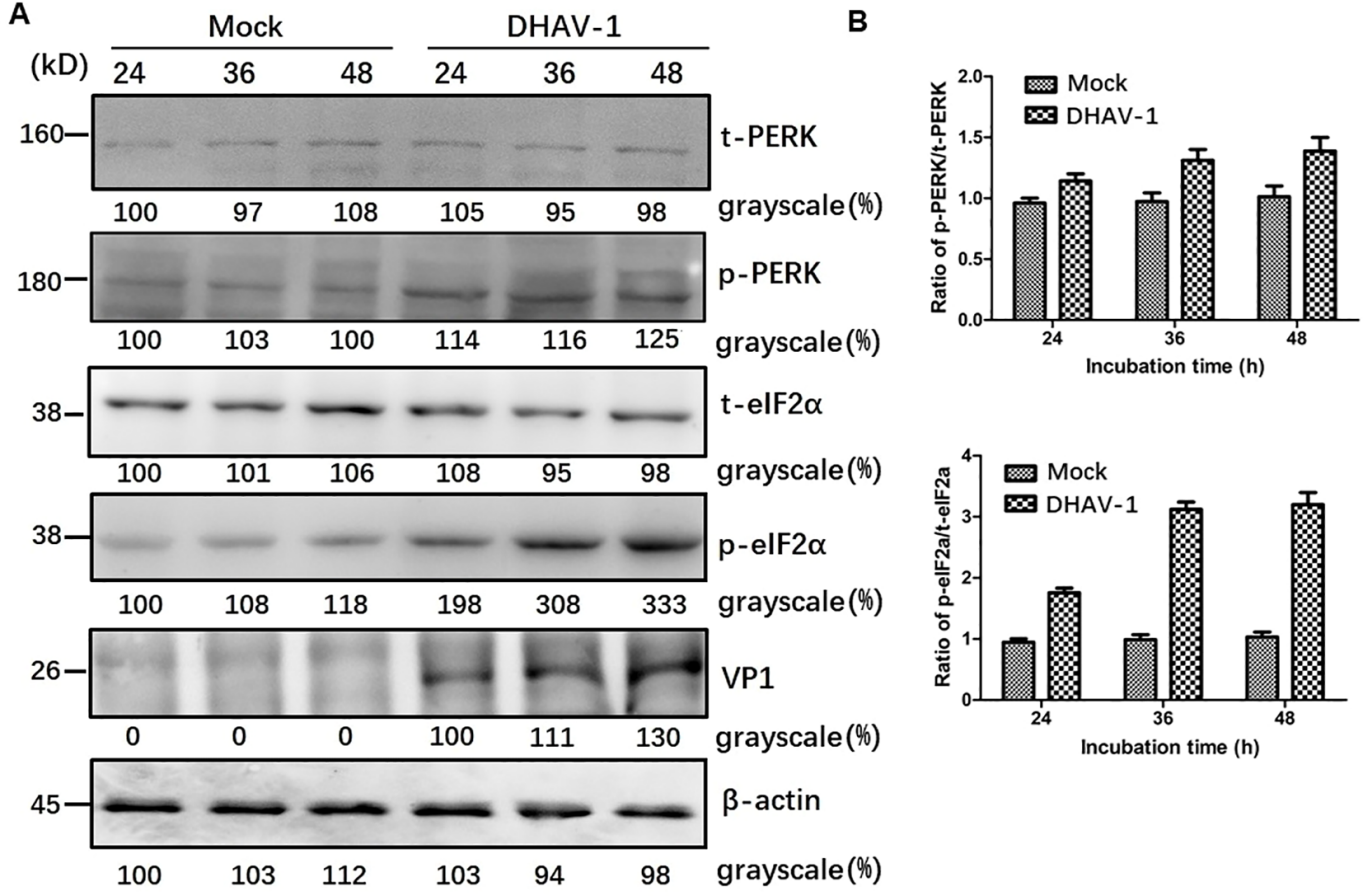
DHAV-1 infection activated the PERK pathway. **(A)** The t-PERK, p-PERK, t-eIF2α, p-eIF2α, VP1 and β-actin expressions in mock-infected cells and DHAV-1-infected cells were generally analyzed using western blotting assay, showing p-eIF2α level was upregulated with the replication of in DHAV-1. **(B)** Compared with mock-infected group, the ratios of p-PERK to t-PERK, and p-eIF2α to t-eIF2α in infected cells were both upregulated from 24 to 48 hpi.

The UPR encompasses three distinct arms involving the activation of ATF-6, PERK and IRE1, respectively. It is well known that ERS and PKR are two major pathways that regulate eIF2a during virus infection (42, 43), and p-eIF2α inhibits global protein translation and thereby reduces protein folding load during ERS. To determine which arm of the URPs is activated during DHAV-1 infection, we conducted further investigation and analyzed eIF2α phosphorylation in lysates from DHAV-1-infected cells using western blotting analysis. The results revealed that the p-eIF2α level was upregulated in DHAV-1-infected cells, while total eIF2α (t-eIF2α) protein level was unchanged (**Figure 2**), suggesting that DHAV-1 infection activated PERK arm of the UPR, resulting in the phosphorylation of eIF2α. In addition, the kinetics of eIF2α phosphorylation induction coincided with the expression level of DHAV-1 VP1 protein, indicating that ERS was involved in the process of DHAV-1 replication.

### DHAV-1 infection induces proapoptotic CHOP expression

**Figure 3:**
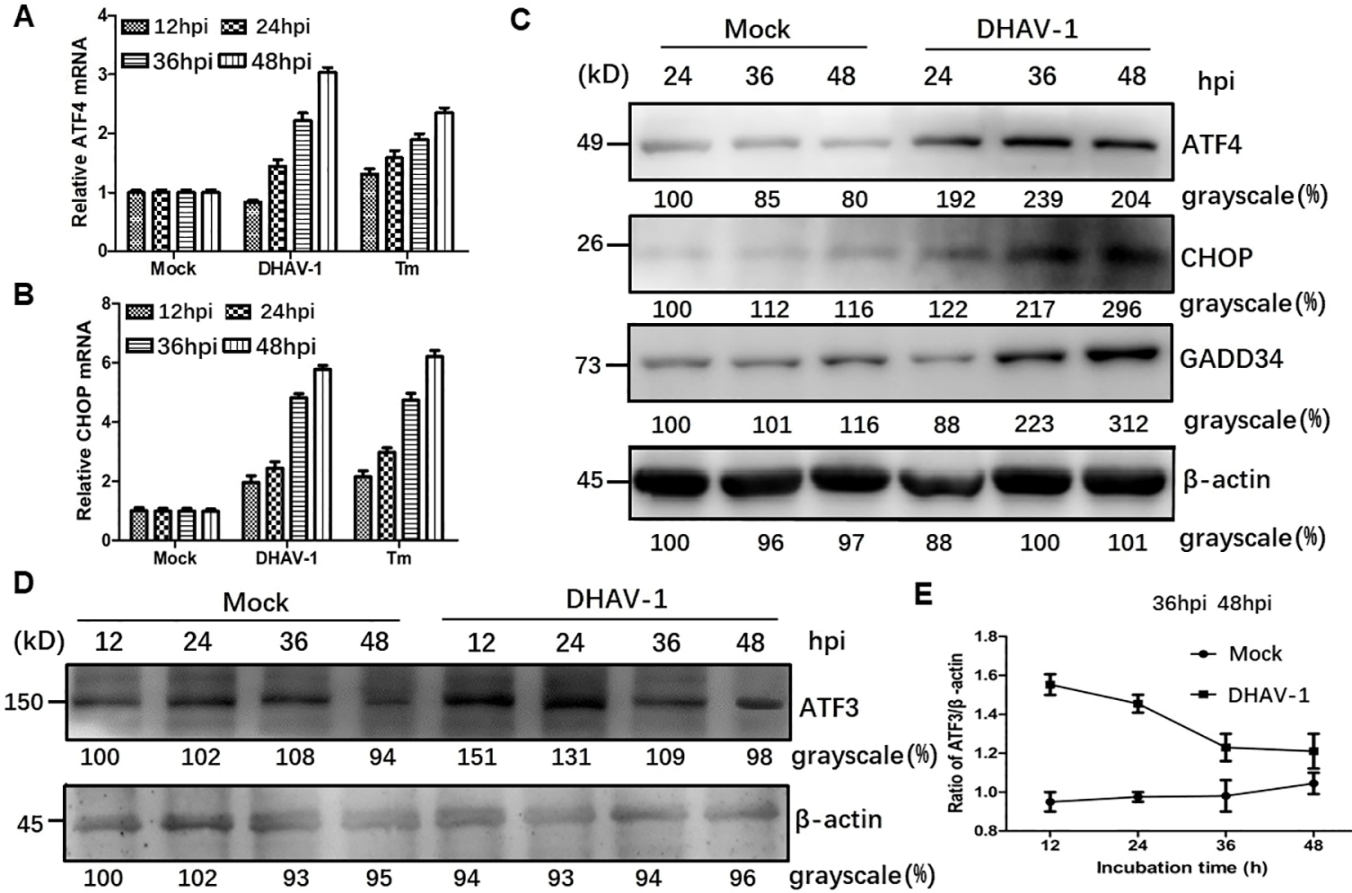
DHAV-1 infection activated the eIF2α-ATF4-CHOP pathway. **(A, B)** Compared with mock-infected group, the ATF4 and CHOP mRNA levels in DHAV-infected cells were persistently increased from 24 to 48 hpi. Tunicamycin (Tm) was respectively used as positive controls. **(C)** Western blotting analysis revealed a consistent and sustained elevation in the protein expression levels of ATF4, CHOP, and GADD34 during DHAV-1 infection. **(D)** ATF3 exhibited upregulation in response to DHAV-1 infection and showed significantly increases at 12 and 24 hpi. **(E)** The ration of ATF3 to β-actin in DHAV-infected cells shows a marked decrease, while ration in mock-infected groups shows a steady rise from 24 to 48 hpi.

Activation of PERK and subsequent phosphorylation of eIF2α selectively promote the transcripti*on of ATF4*, which in turn upregulate *CHOP* and GADD34, leading to apoptosis. To investigate whether the downstream proteins of p-eIF2α were induced by DHAV-1 infection, we examined the mRNA levels of *ATF4* and *CHOP*, as well as the protein expression of ATF4, CHOP, GADD34, and ATF3. The RT-qPCR results demonstrated persistent increases in *ATF4* and *CHOP* mRNA levels during DHAV-1 infection (**Figures 3A, B**). Furthermore, western blotting analysis revealed a consistent and sustained elevation in the protein expression levels of ATF4, CHOP, and GADD34 during DHAV-1 infection (**Figure 3C**). Notably, as an *ATF4* target gene, *ATF3* exhibited upregulation in response to DHAV-1 infection and showed significantly increases at 12 and 24 hpi, indicating its involvement in the earlier stage of ERS (**Figures 3D, E**).

### DHAV-1 infection activates IRE1 arm of UPR

**Figure 4:**
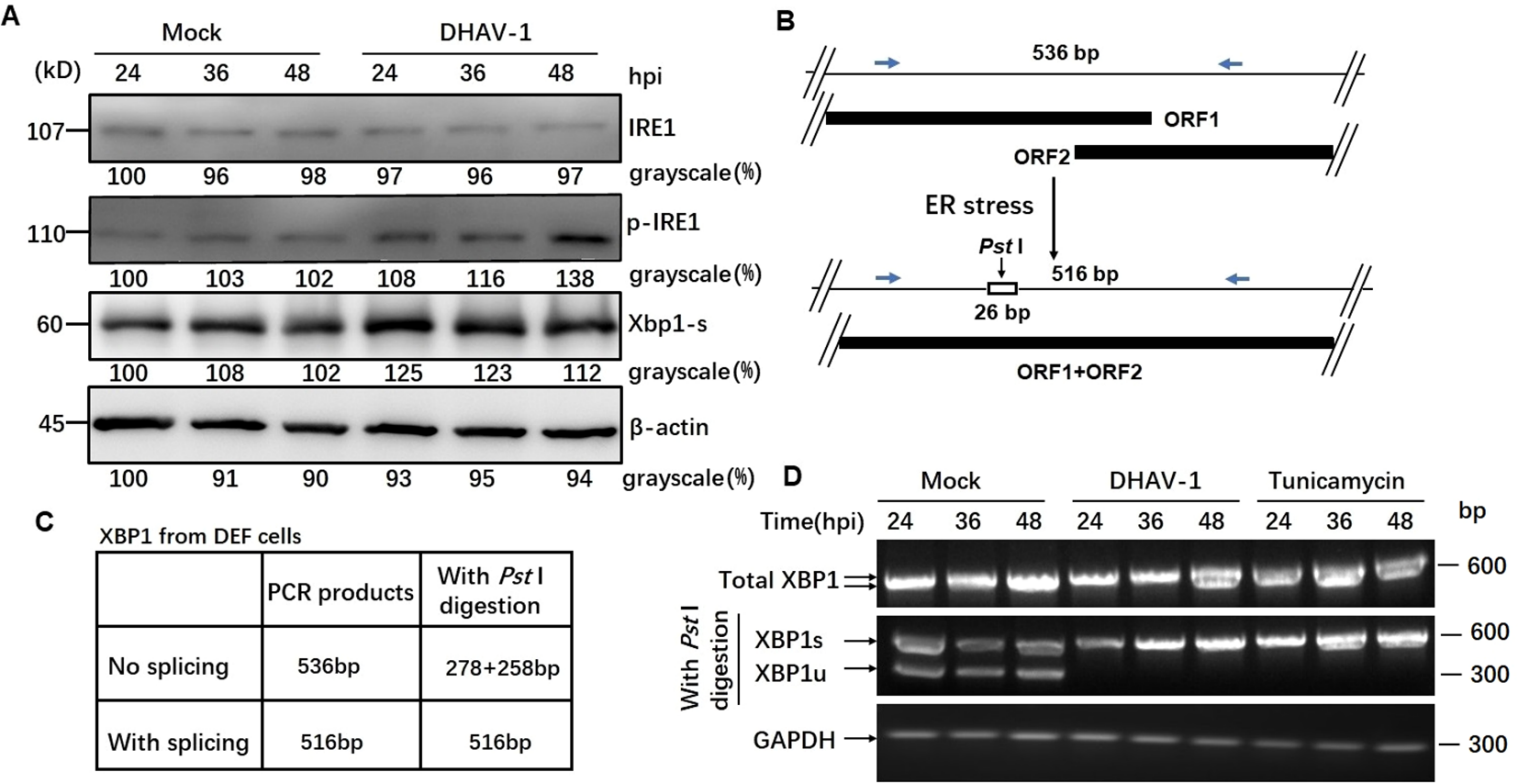
DHAV-1 infection activated the IRE1 pathway. **(A)** Western blotting analysis showed an increased level of phosphorylated IRE1 during DHAV-1 infection, while the total IRE1 expression level remained unchanged. The protein level of the spliced XBP1 (XBP1s) were upregulated particularly at 24 hpi, with a slightly increase observed at 36 and 48 hpi. **(B)** The analysis scheme of XBP1 mRNA splicing: IRE1 endoribonuclease activation leads to the splicing of XBP1 mRNA by removing a 26-bp intron that includes a PstI restriction site. **(C)** The size of PCR-amplified fragments from spliced and un-spliced XBP1 with or without PstI cleavage were listed. **(D)** RT-PCR analysis of XBP1 mRNA splicing: XBP1s mRNA could be detected in DHAV-infected and tunicamycin-treated cells, while un-spliced XBP1 mRNA was identified in the mock-infected cells.

Activation of IRE1 pathway leads to the splicing of *XBP1* mRNA (44). In our study, western blotting results revealed an increased level of phosphorylated IRE1 during DHAV-1 infection, while the total IRE1 expression level remained unchanged (**Figure 4A**). Moreover, the protein level of the spliced XBP1 (XBP1s) were significantly upregulated, particularly at 24 hpi with a slightly increase observed at 36 and 48 hpi (**Figure 4A**).

As known, IRE1 endoribonuclease activation leads to the splicing of *XBP1* mRNA by removing a 26-bp intron that includes a *Pst* I restriction site (**Figure 4B**). To examine the effect of DHAV-1 infection on XBP1 splicing, we performed RT-PCR followed by *Pst* I restriction digestion. Using specific primers, the un-spliced XBP1 fragment was represented by two separate bands of 278 bp and 258 bp, while the presence of a 578 bp indicated the spliced XBP1 (**Figure 4C**). The RT-PCR results revealed that the detection of *XBP1s* mRNA in DHAV-1-infected and tunicamycin-treated cells, while un-spliced *XBP1* mRNA was identified in the mock-infected cells (**Figure 4D**). These results suggested that DHAV-1 infection activated the IRE1-XBP1 pathway under ERS.

To further investigate whether all three ATF6 pathways of the UPR were activated during DHAV-1 infection, we further examined the ATF6 pathway. When the ATF6 was activated, ATF6 was cleaved by transmembrane proteases, resulting in the active N-terminal 50-kDa ATF6 fragment. However, no ATF6 cleavage was observed in DHAV-1-infected cells, indicating that DHAV-1 infection did not active ATF6 pathway (data not shown).

### DHAV-1 infection induces ERS-mediated apoptosis

**Figure 5:**
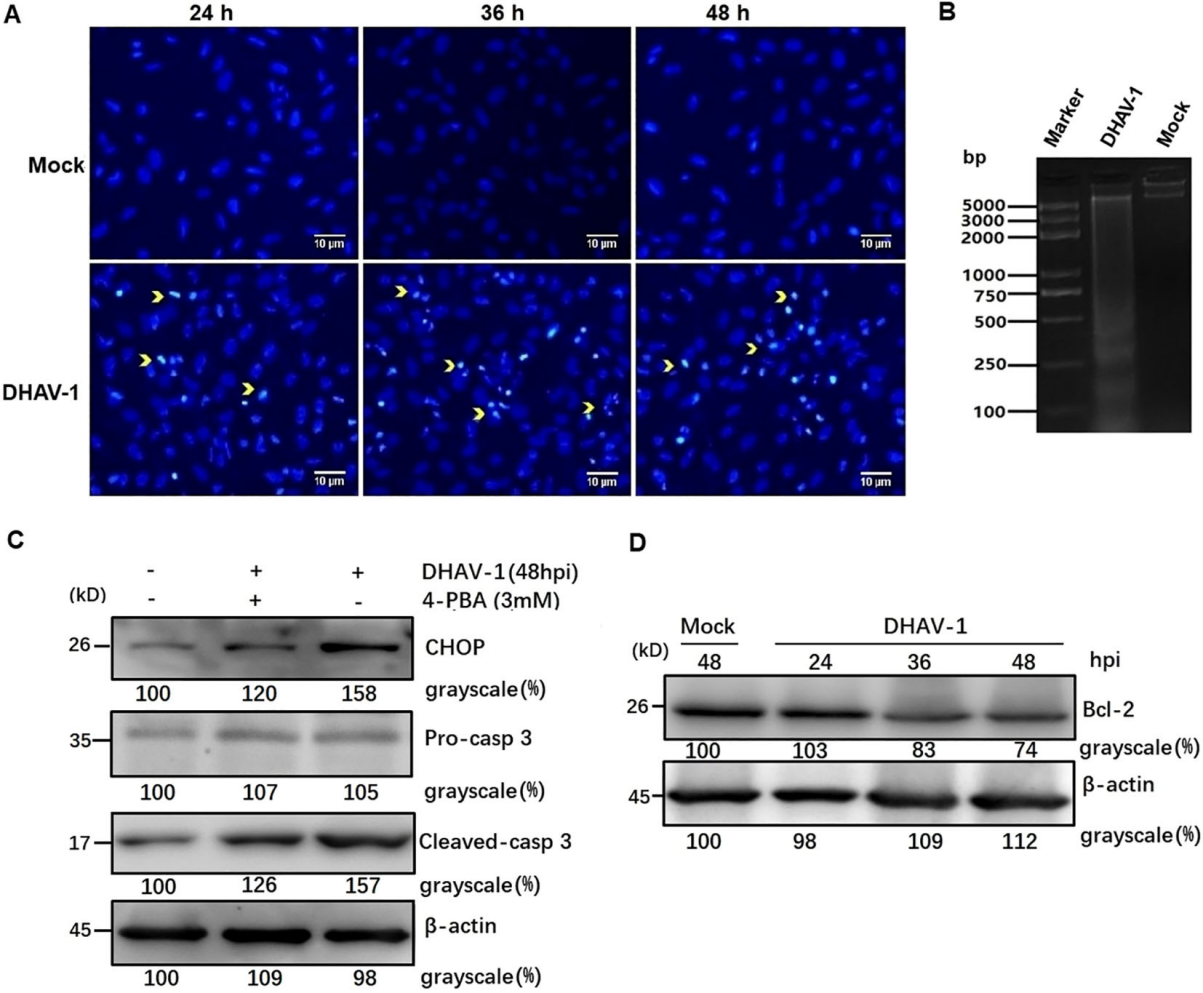
DHAV-1 infection induces CHOP-mediated apoptosis in DEF cells. **(A)** The DHAV-infected cells exhibited condensed nuclei with intense staining compared to mock cells. Yellow arrows indicate condensed nuclei. **(B)** Quick detection of apoptotic DNA ladder in DEF cells with DHAV-1 infection. Lane marker: standard molecular size maker (5 Kb). Lane 2: DAHV-1-infected cells. Lane Mock: Mock-infected cells. **(C)** The upregulation of cleaved caspase-3 was decreased when ERS caused by DHAV-1 was inhibited with 4-PBA treatment. **(D)** The anti-apoptotic protein Bcl-2 was down-regulated from 24 hpi to 48 hpi during DHAV-1 infection.

Nuclear shrinkage and DNA fragmentation are typical characteristics of cell apoptosis. To assess the role of DHAV-1 infection in nuclear morphologic change, we compared chromatin condensation in DHAV-1-infected and mock-infected cells. DHAV-1-infected cells exhibited condensed nuclei with intense staining compared to mock cells (**Figure 5A**). Furthermore, DNA ladder assay demonstrated DNA fragmentation in DEF cells infected with DHAV-1 (**Figure 5B**). These results indicated that DHAV-1 infection induced apoptosis in DEF cells.

To further confirm DHAV-1-induced apoptosis and investigate the relationship between the UPR and apoptosis during DHAV-1 infection, we examined the level of cleaved caspase-3 protein. Western blotting analysis revealed an upregulation of cleaved caspase3, which correlated with the expression level of CHOP (**Figure 5C**). Notably, the upregulated expression levels of caspase-3 and CHOP induced by DHAV-1 were decreased in cells treated with ERS inhibitor 4-PBA (**Figure 5C**). It is well known that CHOP promotes cell death by activating proapoptotic genes and downregulating antiapoptotic genes. In this study, one of the downstream effectors of CHOP, GADD34, was upregulated during DHAV-1 infection (**Figure 4C**). Moreover, anti-apoptotic protein Bcl-2 exhibited a significant decrease from 24 hpi to 48 hpi (**Figure 5D**). These results provide evidence that DHAV-1 infection induced ERS-mediated apoptosis.

### Blocking the UPR reduces DHAV-1-induced apoptosis in DEF cells

**Figure 6:**
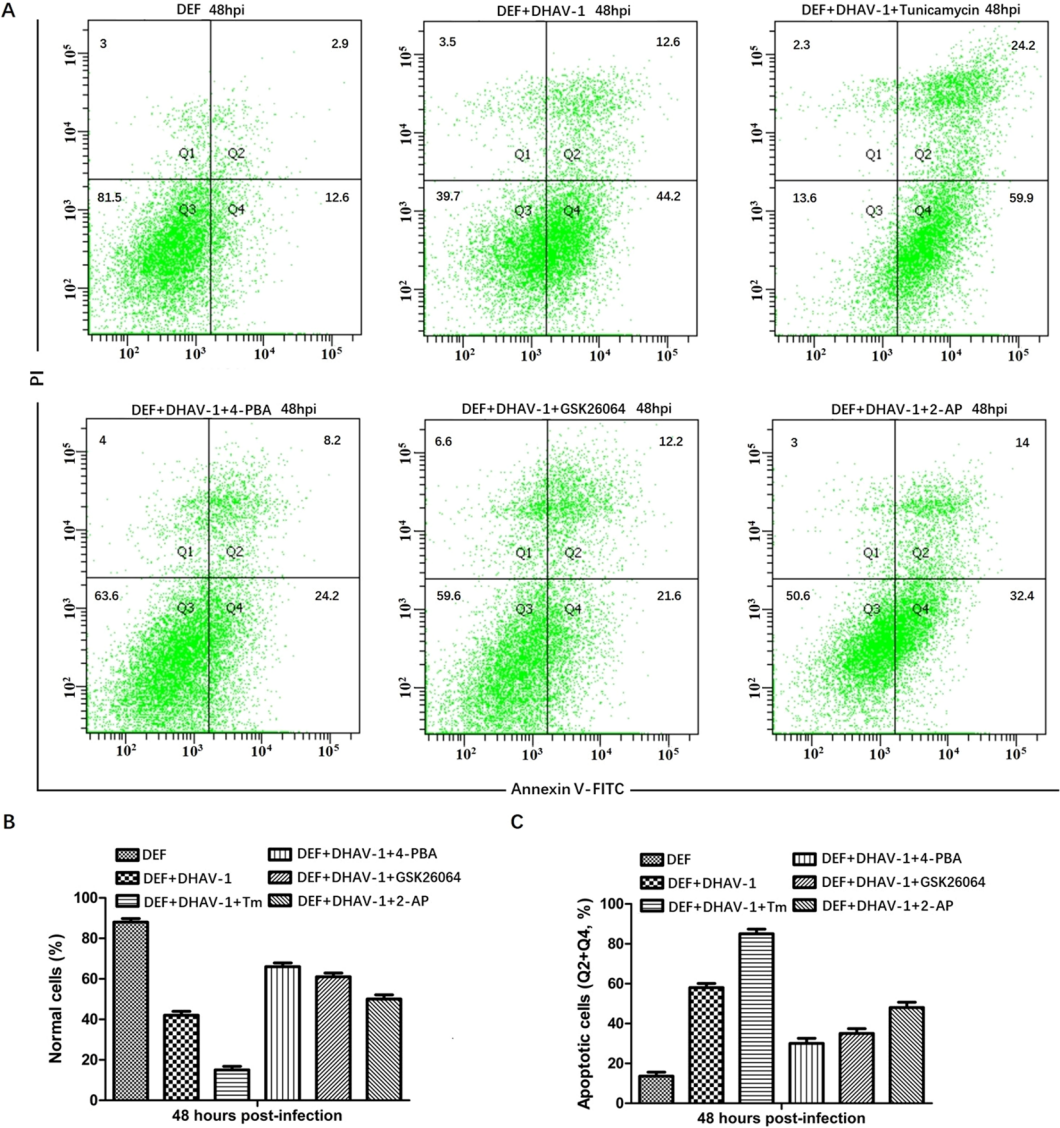
DHAV-1 infection leads to CHOP-induced caspase-3 cleavage. **(A)** The effects of PERK inhibitor GSK2606414 and PKR inhibitor 2-aminopurine (2-AP) on DHAV-1-infected DEF cells apoptosis was analyzed using flow cytometry, tunicamycin and 4-PBA were respectively used as positive and negative controls. The results showed the percentage of apoptotic cells in DHAV-1-infected group was 56.8%, the mock-infected group was 15.5%, the tunicamycin-treated group was 84.1%, the 4-PBA-treated group was 32.4%. **(B)** The percentage of normal cells in different treated groups was consistent with the above flow cytometry results. **(C)** The percentage of apoptotic cells in different treated groups was consistent with the flow cytometry results. All experiments were repeated three times.

The PERK-eIF2α-ATF4-CHOP pathway of UPR plays a vital role in ERS-induced apoptosis. In response to stress signal, the IFN-induced double-stranded RNA-dependent PKR can also phosphorylate the eIF2α (45). Moreover, PKR not only contributes to the early stage of eIF2α phosphorylation but also elicits eIF2α-ATF4-CHOP signaling in ERS-induced apoptosis during viral infection (38, 46). Our proteomic analysis confirmed the high expression of PKR in DHAV-1-infected cells (47). To investigate whether DHAV-1 induces apoptosis through the PERK/PKR-eIF2α-ATF4-CHOP pathway, we first analyzed the effect of PERK inhibitor GSK2606414 and PKR inhibitor 2-aminopurine (2-AP) on DHAV-1-infected DEF cells apoptosis using flow cytometry. Tunicamycin and 4-PBA were used as positive and negative controls, respectively. As shown in **Figure 6A**, DHAV-1 infection increased the percentage of apoptotic cells to 56.8% compared to 15.5% of the mock-infected cells. The percentage of apoptotic cells induced by tunicamycin was 84.1%, whereas the percentage of apoptotic cells treated with 4-PBA was 32.4%. These results indicated that DHAV-1 infection and the ERS inducer tunicamycin promoted DEF cells apoptosis. Moreover, both the PERK inhibitor GSK2606414 and PKR inhibitor 2-AP significantly inhibited DHAV-1-induced apoptosis, with the PERK inhibitor demonstrating a stronger inhibitory effect on DHAV-1-induced apoptosis (**Figures 6B, C**).

### Involvement of PERK/PKR in ERS-mediated apoptosis

**Figure 7:**
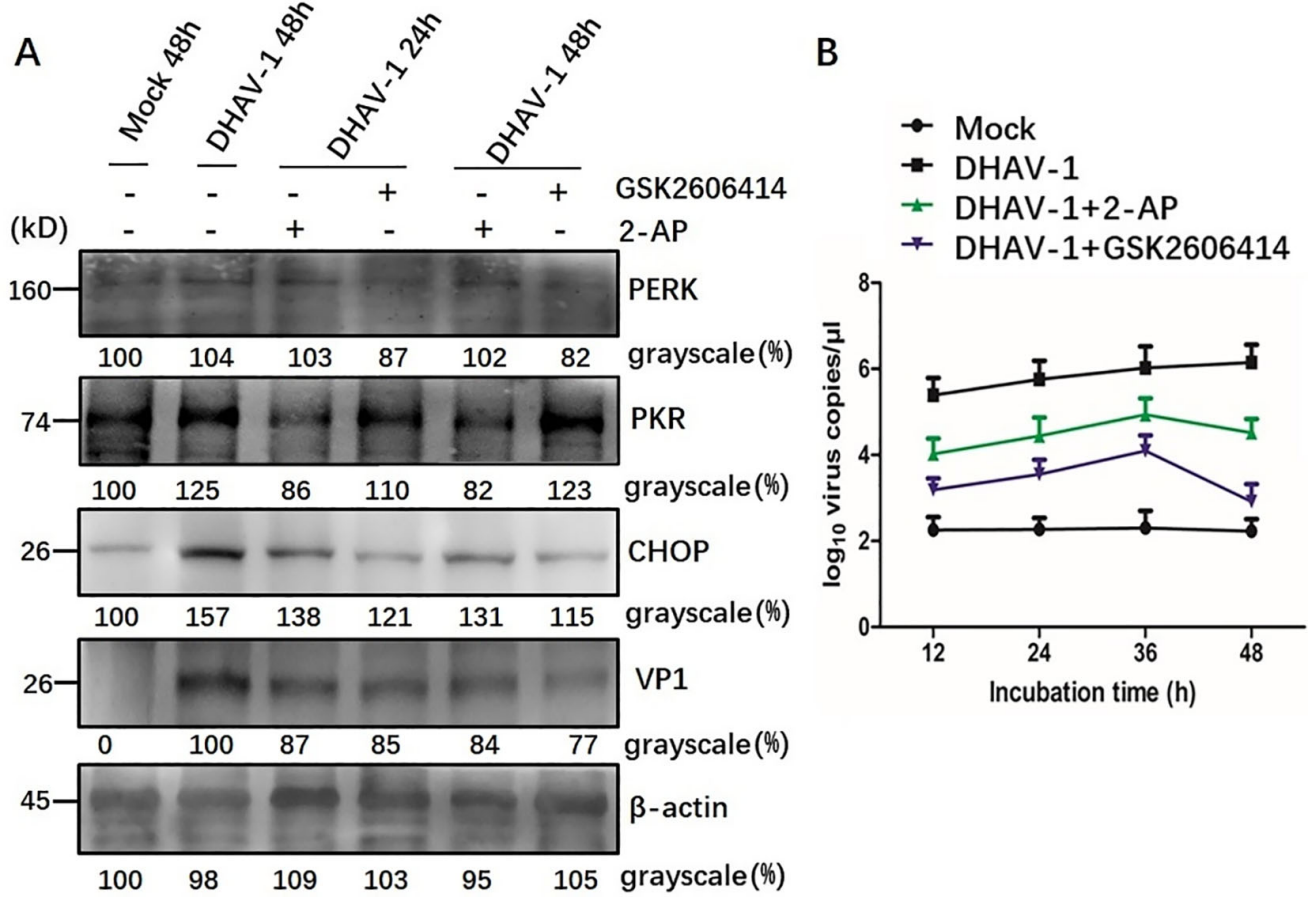
Involvement of PERK/PKR in ERS-mediated apoptosis during DHAV-1 replication. **(A)** Western blotting assay was designed to evaluate the effects of PERK inhibition and PKR inhibition on the ERS-mediated apoptosis: cells treated with GSK2606414 or 2-AP showed a significant reduction in the respective protein level and CHOP protein level compared to the mock-infected cells. A lower expression level of VP1 protein was observed in DHAV-1-infected cells treated with pharmacological intervention. **(B)** The RT-qPCR was designed to evaluate the effects of PERK inhibition and PKR inhibition on DHAV-1 replication: the viral copies in cells treated with GSK2606414 or 2-AP treated showed a significant decrease. Moreover, the virus copies in GSK2606414-treated cells were lower than those in 2-AP treated cells.

Western blotting analysis further revealed that cells treated with GSK2606414 or 2-AP showed a significant reduction in the respective protein level and CHOP protein level compared to the mock-infected cells. Additionally, a lower expression level of VP1 protein was observed in DHAV-1-infected cells treated with pharmacological intervention (**Figure 7A**). This result was further supported by viral copies number analysis using RT-qPCR, which showed significant decrease in viral copy numbers in cells treated with GSK2606414 or 2-AP, indicating that inhibiting apoptosis attenuated the virus replication (**Figure 7B**). Moreover, the virus copy numbers in GSK2606414-treated cells were lower than those in 2-AP treated cells, indicating that PERK pathway had a greater effect on DHAV-1 replication than the PKR pathway. Overall, these results indicated that the PERK/PKR- eIF2α-ATF4-CHOP pathway was involved in the ERS-mediated apoptosis caused by DHAV-1 infection and DHAV-1 replication.

## Discussion

**Figure 8:**
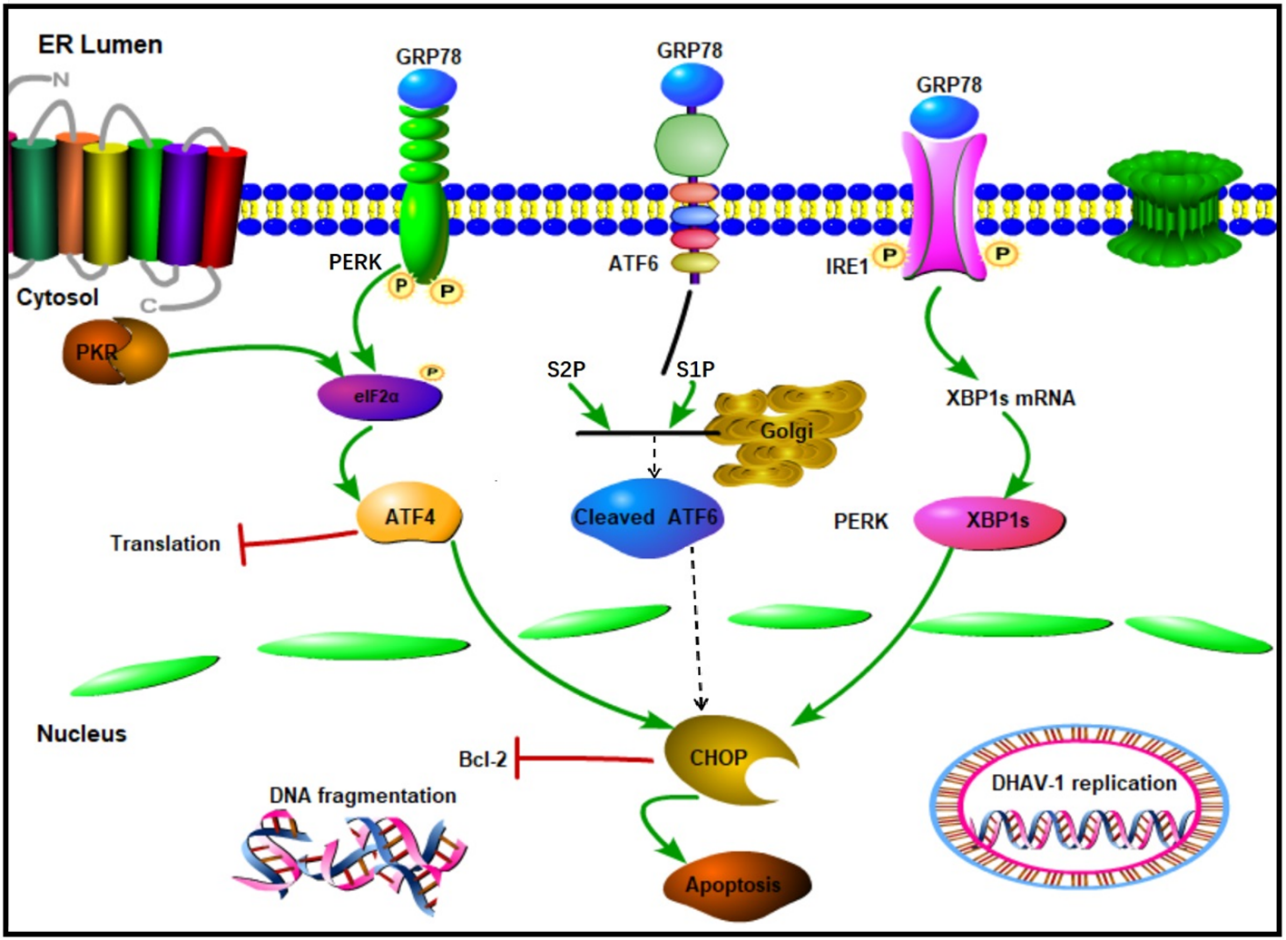
Schematic diagram of UPR caused by DHAV-1 infection. DHAV-1 infection leads to UPR through PERK-eIF2α-ATF4 and IRE1-XBP1s pathways. Both PERK and PKR participate in the regulation of p-eIF2α and ATF4 to modulate apoptosis and viral replication. DHAV-1 infection induces DNA breakage, and CHOP protein exerts its proapoptotic activity by inhibiting Bcl-2. Sharp arrows indicate activation, blunt lines indicate inhibition.

ER is a crucial multifunctional organelle that serves as the site of viral replication and maturation for several viruses (48). During viral infection, a large amount of viral protein synthesis results in the accumulation of unfolded or misfolded proteins in the ER, triggering ERS and activating the UPR (49, 50). Our previously proteomic result also revealed that the activation of ERS-induced autophagy by DHAV-1 (47). However, the relationship between the ERS and apoptosis caused by DHAV-1 has not yet to be fully elucidated. In this study, we identified the activation of UPR process and investigated the relationship between UPR and apoptosis caused by DHAV-1. We found that the PERK/PKR pathway plays a crucial role in ERS-induced apoptosis and DHAV-1 replication. This effect is achieved by inducting the proapoptotic transcription factor CHOP, which ultimately activates caspase-3 and promotes cell apoptosis (**Figure 8**).

GRP78 is the master regulator of the UPR pathway and governs the UPR activation (51). Our current study, using western blotting and TEM, demonstrated significant upregulation of GRP78 and expansion of ER membrane in DHAV-1-infected cells (**Figure 1**), indicating that DHAV-1 activation of ERS. The PERK pathway, one of the most important arms of UPR, phosphorylates eIF2α, leading to the inhibition of general protein translation but the selectively promotion of ATF4 translation during ERS (36). Moreover, ATF4 plays a crucial role in adapting to stresses by regulating the transcription of numerous genes (52). Our western blotting result showed that eIF2α was phosphorylated along with the PERK activation caused by DHAV-1, resulting in the upregulation of ATF4, ATF3, and CHOP (**Figures 2**, **3**). Further research results showed that DHAV-1 infection also activated the IRE1 pathway (**Figure 4**), and the ATF6 pathway remained inactive. Our study provides a notable example of how a virus exploits the UPR pathway to cope with ERS induced by DHAV-1 infection. It has been reported that many viruses have evolved strategies to modulate UPR for their own replication (53–55). Furthermore, we observed that the activation of ERS could enhance DHAV-1 replication, whereas inhibition of ERS with 4-PBA could significantly reduce viral replication (**Figure 1**). Thus, it is conceivable that DHAV-1 infection induces ERS and modulates the UPR, leading to a shutdown of host protein synthesis and enhancement of viral protein translation, ultimately benefiting viral replication.

Viral infection can induce various forms of cell death. Previous studies have reported that DHAV-1 can trigger pyroptosis; however, apoptosis appears to be more common in DHAV-1 infections. Cell apoptosis has been observed in DHAV-infected ducklings, indicating its potential significance in DHAV-1 infection. Apoptosis is characterized by the translocation of membrane phosphatidylserine (PS) from the inner side of the plasma membrane to the surface. Annexin V, which has a high affinity for PS, can be used to stain cells for Annexin V-FITC/PI analysis (56). Additionally, apoptosis can be identified by ladder pattern of 180-200 bp fragment resulting from DNA cleavage (57). In our study, we demonstrated an increased apoptosis rate caused by DHAV-1 infection from 12 hpi to 48 hpi, along with the formation of a DNA ladder in DHAV-1-infected cells (**Figure 5**).

The UPR is initially a pro-survival signal aiming at restoring ER homeostasis. However, under persistent ERS, it can switch to a pro-apoptotic in model (58). The PERK and IRE1 arms of UPR, during persistent ERS, can suppress the activity of antiapoptotic proteins and activate the expression of proapoptotic proteins (59). In our study, DHAV-1 infection upregulation the expression of CHOP, which is recognized as one of the proapoptotic proteins induced by ATF4 and ATF3 (35). It has been reported that there is a close relationship between ER and mitochondria, and ERS can activate caspase-3 leading to apoptosis (60). Western blotting results revealed that the activation and cleavage of caspase-3 in DHAV-1-infected cells, while the expression level of cleaved-caspase-3 decreased with the inhibition of ERS (**Figure 5**). The ATF4-CHOP-mediated transactivation of the Bcl-2 family is involved in ERS-induced cell apoptosis pathway (61, 62). In our study, the anti-apoptosis protein Bcl-2 was down-regulated in DHAV-1-infected cells, suggesting that cell apoptosis caused by DHAV-1 is regulated.

CHOP is a well-known gene that facilitate ERS-induced apoptosis, and its regulation involves a complex mechanism. It has been reported that the upregulation of CHOP is mediated by both the PERK-eIF2α-ATF4 pathway and the PKR-eIF2α-ATF4 pathway (63). In this study, the expression level of CHOP decreased when PERK and PKR were inhibited by GSK26064 and 2-AP (**Figure 6**), which is consistent with CHOP-mediated apoptosis caused by avian infectious bronchitis virus (63). Importantly, we observed a significant decrease in the VP1 protein at 48 hpi, which aligns with RT-qPCR results (**Figure 7**). These findings suggested that the PKR pathway synergistically interacts with the PERK pathway in regulating of ERS-induced apoptosis caused by DHAV-1, thereby contributing DHAV-1 replication. Similar apoptotic regulation strategies have been reported in various virus families during their replication process, such as West Nile virus, BVDV2, HCV, arbovirus, influenza virus, and HIV (64–67).

In conclusion, this report provides evidence of the induction of UPR signaling cascades, the involvement of the PERK/PKR-eIF2α-ATF4-CHOP pathway, and the underlying mechanism of ERS-induced apoptosis caused by DHAV-1 infection. This work provides new insights into DHAV induced apoptosis and the regulation mechanism that benefits viral infection. Furthermore, modulators of the UPR, such as GSK2606424, which inhibits viral replication, may serve as novel therapeutic targets for controlling DHAV infection and pathogenesis.

## Data Availability

The original contributions presented in the study are included in the article/supplementary material. Further inquiries can be directed to the corresponding author.
